# Survival benefits of radiotherapy in locally advanced unresectable and metastatic pancreatic cancer: a single-institution cohort and SEER database analysis

**DOI:** 10.3389/fonc.2024.1473251

**Published:** 2024-09-16

**Authors:** Bi-Yang Cao, Le-Tian Zhang, Chen-Chen Wu, Jing Wang, Lin Yang

**Affiliations:** ^1^ Department of Medical Oncology, National Cancer Center/National Clinical Research Center for Cancer/Cancer Hospital, Chinese Academy of Medical Sciences and Peking Union Medical College, Beijing, China; ^2^ Department of Radiation Oncology, First Medical Center of Chinese People’s Liberation Army General Hospital, Beijing, China

**Keywords:** radiotherapy, chemotherapy, locally advanced, metastatic, pancreatic cancer, Surveillance, Epidemiology, and End Results (SEER), overall survival

## Abstract

**Background:**

Chemotherapy (CT) remains the primary treatment for locally advanced unresectable pancreatic cancer (LAUPC) and metastatic pancreatic cancer (MPC). The role of radiotherapy (RT) in these conditions remains unclear. This study compares the outcomes of CT alone versus CT combined with RT (combined-modality therapy [CMT]) in LAUPC and MPC patients.

**Materials and methods:**

We conducted a retrospective analysis of LAUPC and MPC patients treated with either CT or CMT from a single institution and Surveillance, Epidemiology, and End Results (SEER) database. Kaplan-Meier curves and Cox hazards models evaluated the association between treatment modalities and overall survival (OS). Propensity score matching (PSM) ensured balanced comparisons. Landmark analysis addressed immortal time bias. Subgroup analyses were based on clinical characteristics. eXtreme Gradient Boosting (XGBoost) and Shapley Additive Explanations (SHAP) assessed outcome prediction and influence of significant predictors.

**Results:**

The study included 102 patients receiving CMT and 155 receiving CT at single institution, along with 1733 CMT and 9310 CT patients from the SEER dataset. In the single-institution cohort, CMT showed superior survival compared to CT both before (median OS: 20.5 vs. 11.5 months, hazard ratio [HR]: 0.47, 95% CI: 0.34-0.65, P=0.001) and after PSM (median OS: 22.2 vs. 11.8 months, HR: 0.49, 95% CI: 0.30-0.79, P=0.003). Multivariate analyses confirmed that CMT was independently associated with improved OS both before (HR: 0.54, 95% CI: 0.38-0.77, P=0.001) and after PSM (HR: 0.45, 95% CI: 0.27-0.73, P=0.001). Landmark analysis indicated better OS for patients receiving CMT compared to CT alone. Subgroup analysis revealed an OS benefit for CMT across most subgroups. SHAP value analysis indicated that CMT was the most significant contributor to survival outcomes. SEER database validation confirmed these findings.

**Conclusions:**

This study demonstrates that CMT significantly improves OS in LAUPC and MPC patients compared to CT alone. Integrating RT with CT could be beneficial for treating LAUPC and MPC.

## Introduction

Pancreatic cancer (PC) is among the most lethal malignancies, with a poor prognosis and limited treatment options. The standard treatments for locally advanced unresectable pancreatic cancer (LAUPC) and metastatic pancreatic cancer (MPC) primarily involve chemotherapy (CT). Despite advances in chemotherapy regimens, overall survival (OS) rates remain dismal (1).

For LAUPC, conventional strategies include CT and chemoradiotherapy (CRT).Numerous randomized controlled trials (RCTs) (2–6) and meta-analyses (7) have compared CRT with CT alone, yet the optimal therapy remains uncertain. Among relevant randomized controlled studies (2–6), some studies (3, 5) have found that CMT is superior to CT, while others (2, 4, 6) found no survival benefits from CMT. No consensus has been reached regarding the treatment of LAUPC. The National Comprehensive Cancer Network (NCCN) suggests various treatments, including CT alone, CT followed by CRT or stereotactic body radiotherapy (SBRT), and CRT or SBRT alone (8). In contrast, the European Society of Medical Oncology (ESMO) recommends CT alone as the primary treatment, with CRT playing a secondary role (9).

Systemic therapy is the primary palliative treatment for MPC, which has a 5-year OS rate of just 3% (10). The local tumor burden significantly contributes to morbidity and mortality, often causing gastrointestinal distress, biliary complications, and intense pain due to adjacent organ involvement (11). Investigating novel combinations of systemic therapies, such as FOLFIRINOX and gemcitabine with nab-paclitaxel, has shown incremental improvements in clinical outcomes for MPC patients (12, 13). Additionally, advancements in local tumor control offer promise for improving clinical outcomes and quality of life (14–16). Specially, compared with conventionally fractionated external beam radiotherapy, SBRT delivers higher biologically effective doses of radiation on the tumor with less impact on adjacent organs over fewer sessions, allowing improved local control (17). SBRT has shown significant potential in the treatment of MPC, particularly for patients with oligometastases, providing effective local control, and alleviating symptoms while maintaining patient quality of life (18, 19). The addition of radiotherapy (RT) to CT, known as combined-modality therapy (CMT), has shown conflicting results in LAUPC and MPC. While the use of RT has been demonstrated to improve local control, its ability to translate this improvement into a long-term survival benefit remains controversial. This uncertainty highlights the need for further investigation into the survival benefits of CMT compared to CT alone. To address these uncertainties, we conducted a comprehensive analysis using both single-institution data and the Surveillance, Epidemiology, and End Results (SEER) database. This dual approach allows for a robust comparison of treatment outcomes, accounting for variations in patient demographics and clinical characteristics. By leveraging large-scale population data and detailed clinical records, we aim to provide clearer insights into the role of RT in managing LAUPC and MPC.

Machine learning (ML) has become an increasingly prominent tool in clinical research, offering robust capabilities for managing extensive and heterogeneous datasets, uncovering intricate patterns, and predicting complex outcomes (20). Traditionally, the Cox proportional hazards regression model and the Kaplan-Meier estimator have been the primary methods employed to identify significant risk factors and estimate survival probabilities. However, recent advancements in ML models for survival analysis have addressed limitations inherent in conventional approaches, particularly when the assumption of constant proportional hazards over time is violated.

Among these, the eXtreme Gradient Boosting (XGBoost) algorithm stands out as an optimized distributed gradient boosting library designed for efficiency, flexibility, and portability. XGBoost implements machine learning algorithms within the Gradient Boosting framework, offering fast and accurate solutions to many data science problems.

Given the ongoing uncertainties and mixed results regarding the survival benefits of adding RT to CT in the treatment of LAUPC and MPC, our study seeks to provide a clearer understanding of the role of CMT. By integrating advanced machine learning techniques, such as the XGBoost algorithm, alongside traditional statistical methods, we aim to offer more precise predictions of patient outcomes and identify key prognostic factors. This comprehensive approach, utilizing both single-institution and SEER database data, allows us to address the variability in treatment effects observed across different patient populations. Ultimately, our findings will contribute to the ongoing debate on the efficacy of CMT and help guide future clinical decision-making and research efforts in the management of PC.

## Methods

### Study design

This study utilizes two distinct datasets, a single-institution cohort and the SEER database, to investigate the survival benefits of CMT compared to CT alone in patients diagnosed with LAUPC and MPC. The dual approach enables a robust comparison of treatment outcomes across diverse patient populations. The study’s primary endpoint was the OS, measured from the initial diagnosis of PC to either death from any cause or the last follow-up.

### Single-institution cohort

**Figure 1:**
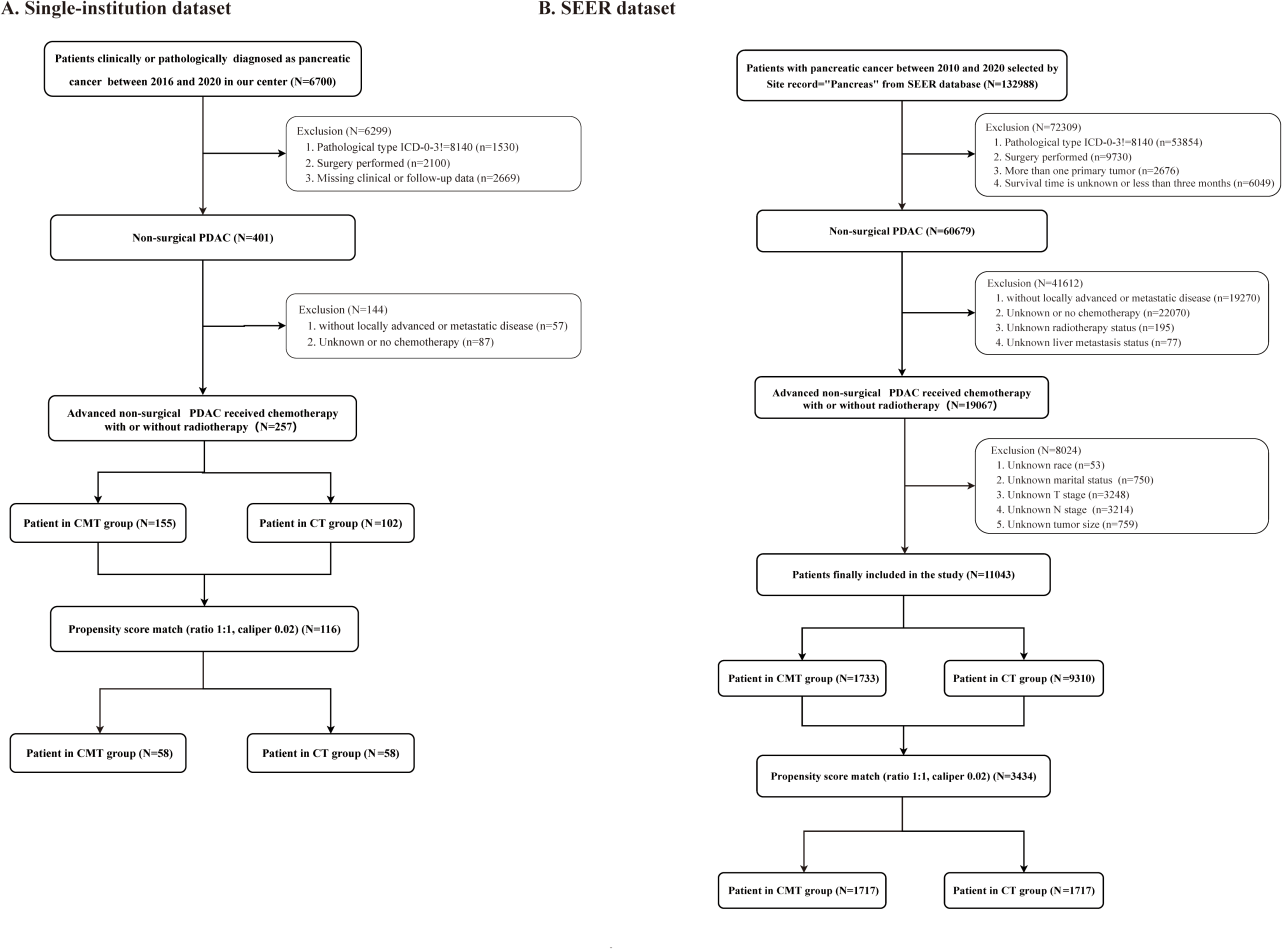
Study flow diagram. **(A)** Single-institution cohort; **(B)** Surveillance, Epidemiology, and End Results (SEER) cohort.

We retrospectively analyzed data from patients diagnosed with LAUPC and MPC at our institution between 2016 and 2020. The following were the key inclusion criteria: (1) age ≥18 years; (2) a confirmed PC diagnosis, either clinically (21) or pathologically; (3) no prior surgery; (4) with locally advanced unresectable or metastatic disease; (5) receiving CT; (6) with clear RT status; (7) survival time ≥3 months; (8) complete clinical and follow-up information. The exclusion criteria were as follows: (1) previous surgery; (2) potentially operable disease with comorbidities preventing surgery (stage I-II); (3) not receiving CT; (4) unclear RT status; (5) incomplete or missing clinical and follow-up information; (6) survival time <3 months; and (7) with a history of other types of cancer within the previous 5 years. The detailed patient selection process is shown in **Figure 1A**. Data collected included demographics, clinical characteristics, treatment details, and survival outcomes.

All patients received CT. The CT regimens followed the systemic therapy for advanced PC as delineated in the NCCN guidelines (8). Various treatment regimens were utilized, such as FOLFIRINOX, gemcitabine combinations, albumin-bound paclitaxel combinations, fluoropyrimidine combinations, as well as single-agent therapies. RT was administered to target primary pancreatic tumors and/or metastatic lesions. In single-institution cohort, 102 patients received CMT. Among them, 76 patients underwent RT targeting the primary pancreatic tumor, 16 received RT for both primary pancreatic tumor and metastatic lesions, and 10 received RT specifically for metastatic lesions. The RT delivery was delivered according to previously described methods (22). Detailed information regarding the CT regimens and RT delivery are shown in **Supplementary Table S1**.

The study adhered to the Declaration of Helsinki and received ethical approval by the Ethics Committee of the First Medical Center of Chinese PLA General Hospital (approval number: S2024-015-01), with patient consent waived due to the retrospective design and no direct patient involvement.

### SEER database analysis

Data from the SEER database was extracted for patients diagnosed with LAUPC and MPC from 2010 to 2020. Inclusion criteria mirrored those of the single-institution cohort. The detailed patient selection process is shown in **Figure 1B**. Variables collected included age, gender, tumor stage, treatment modalities, and survival outcomes. Detailed information regarding the CT regimens and RT delivery in the SEER database is unavailable. No ethical approval or informed consent was required for SEER database analysis due to the use of publicly available SEER data.

### Statistical methods

#### Propensity score matching

Propensity score matching (PSM) was employed to minimize selection bias by matching patients in the CMT and CT groups based on baseline characteristics. To maximally inform the propensity of the dependent variable, all baseline characteristics variables available from the single-institution cohort and SEER database except for the use of treatment were included in the propensity score model. This method ensures comparability between treatment groups. Group baseline characteristics were matched by PSM utilizing nearest-neighbor matching with a 0.02 caliper for 1:1 pairing to minimize selection bias. The standardized mean difference (SMD) method assessed the balance of covariates, with an SMD under 10% indicating a significant balance.

### Machine learning with XGBoost

Under the Gradient Boosting framework, eXtreme Gradient Boosting (XGBoost) is a tree-based ensemble machine learning algorithm (23). This technique was built on creating multiple classifiers, and as such, it regularizes overfitting and outputs good performance in both regression and classification. We utilized the XGBoostalgorithmto predict survival outcomes and identify significant prognostic factors of patients with LAUPC and MPC in two cohorts. This statistical technique was selected for its capacity to produce more stable and accurate average predictions. Following the modeling process, we further enhanced interpretability through the application of Shapley Additive Explanations (SHAP).

### Landmark analysis

To address immortal time bias, we employed a landmark analysis (24). Immortal time bias arises when there is a period during which the outcome (e.g., death) cannot occur. In our study, patients must survive long enough to receive CMT, creating a potential bias if this period is not properly accounted for. We defined a landmark time point at 6 months post-treatment initiation. Only patients who survived beyond this landmark were included in the analysis, ensuring that all patients had an equal opportunity to receive CMT or CT alone, thus mitigating the bias introduced by differential survival times before treatment initiation.

### Survival analysis

Survival analyses were analyzed with the Kaplan–Meier method and differences assessed using the log-rank test. The Cox proportional hazards model was then utilized for multivariable analysis to determine hazard ratios (HR) and their 95% confidence intervals (CI).

All statistical analyses were performed using R software version 4.0.4 (http://www.r-project.org). The PSM was conducted using the “MatchIt” package, landmark analysis using the “jskm” package, and XGBoost modeling using the “xgboost” package in in R software.

## Results

### Analyses using the single-institution cohort

#### Clinicopathologic features

A total of 257 patients who satisfied the inclusion criteria were recruited from the single institution between January 2016 and December 2020. They were categorized based on the treatment received, with 102 patients in the CMT group and 155 patients in the CT group before PSM. After PSM, each treatment group consisted of 58 patients. Demographic and clinical details before and after PSM are outlined in **Table 1**. Before PSM, the CMT group had a higher proportion of elderly patients, primary tumors located in the head of the pancreas, T4 disease, stage III disease, absence of liver metastasis, and a lower likelihood of undergoing immunotherapy and targeted therapy than the CT group. After PSM, all baseline characteristics were well-balanced between the CMT and CT groups.

**Table 1:** Patient clinicopathologic characteristics by treatment groups in single-institution dataset before and after PSM.

Variables	Before PSM n (%) of patients (n = 257)			After PSM, n (%) of patients (n = 116)		
	CT	CMT	p	CT	CMT	p
n	155	102		58	58	
Age (years)						
≤55	96 (61.9)	43 (42.2)	0.003	30 (51.7)	29 (50.0)	1
>55	59 (38.1)	59 (57.8)		28 (48.3)	29 (50.0)	
Sex						
Male	99 (63.9)	64 (62.7)	0.959	33 (56.9)	35 (60.3)	0.85
Female	56 (36.1)	38 (37.3)		25 (43.1)	23 (39.7)	
Site						
Head	61 (39.4)	60 (58.8)	0.003	30 (51.7)	27 (46.6)	0.71
Body/tail	94 (60.6)	42 (41.2)		28 (48.3)	31 (53.4)	
Size (cm)						
≤4	73 (47.1)	57 (55.9)	0.211	24 (41.4)	29 (50.0)	0.456
>4	82 (52.9)	45 (44.1)		34 (58.6)	29 (50.0)	
CA19-9 (U/mL)						
≤350	75 (48.4)	53 (52.5)	0.609	34 (58.6)	25 (43.9)	0.162
>350	80 (51.6)	48 (47.5)		24 (41.4)	32 (56.1)	
T stage						
T1	8 (5.2)	5(4.9)	<0.001	2 (3.4)	1 (1.7)	0.951
T2	38 (24.5)	12 (11.8)		7 (12.1)	7 (12.1)	
T3	50 (32.3)	16 (15.7)		12 (20.7)	12 (20.7)	
T4	59 (38.1)	69 (67.6)		37 (63.8)	38 (65.5)	
N stage						
N0	51 (32.9)	57 (55.9)	<0.001	26 (44.8)	23 (39.7)	0.707
N+	104 (67.1)	45 (44.1)		32 (55.2)	35 (60.3)	
Stage						
III	46 (29.7)	67 (65.7)	<0.001	33 (56.9)	34 (58.6)	1
IV	109 (70.3)	35 (34.3)		25 (43.1)	24 (41.4)	
Liver metastasis						
No	64 (41.3)	73 (71.6)	<0.001	36 (62.1)	36 (62.1)	1
Yes	91 (58.7)	29 (28.4)		22 (37.9)	22 (37.9)	
Immunotherapy						
No	54 (34.8)	61 (59.8)	<0.001	23 (39.7)	25 (43.1)	0.85
Yes	101 (65.2)	41 (40.2)		35 (60.3)	33 (56.9)	
Targeted therapy						
No	85 (54.8)	63 (61.8)	0.332	39 (67.2)	30 (51.7)	0.13
Yes	70 (45.2)	39 (38.2)		19 (32.8)	28 (48.3)	

#### Oncologic outcomes

**Figure 2:**
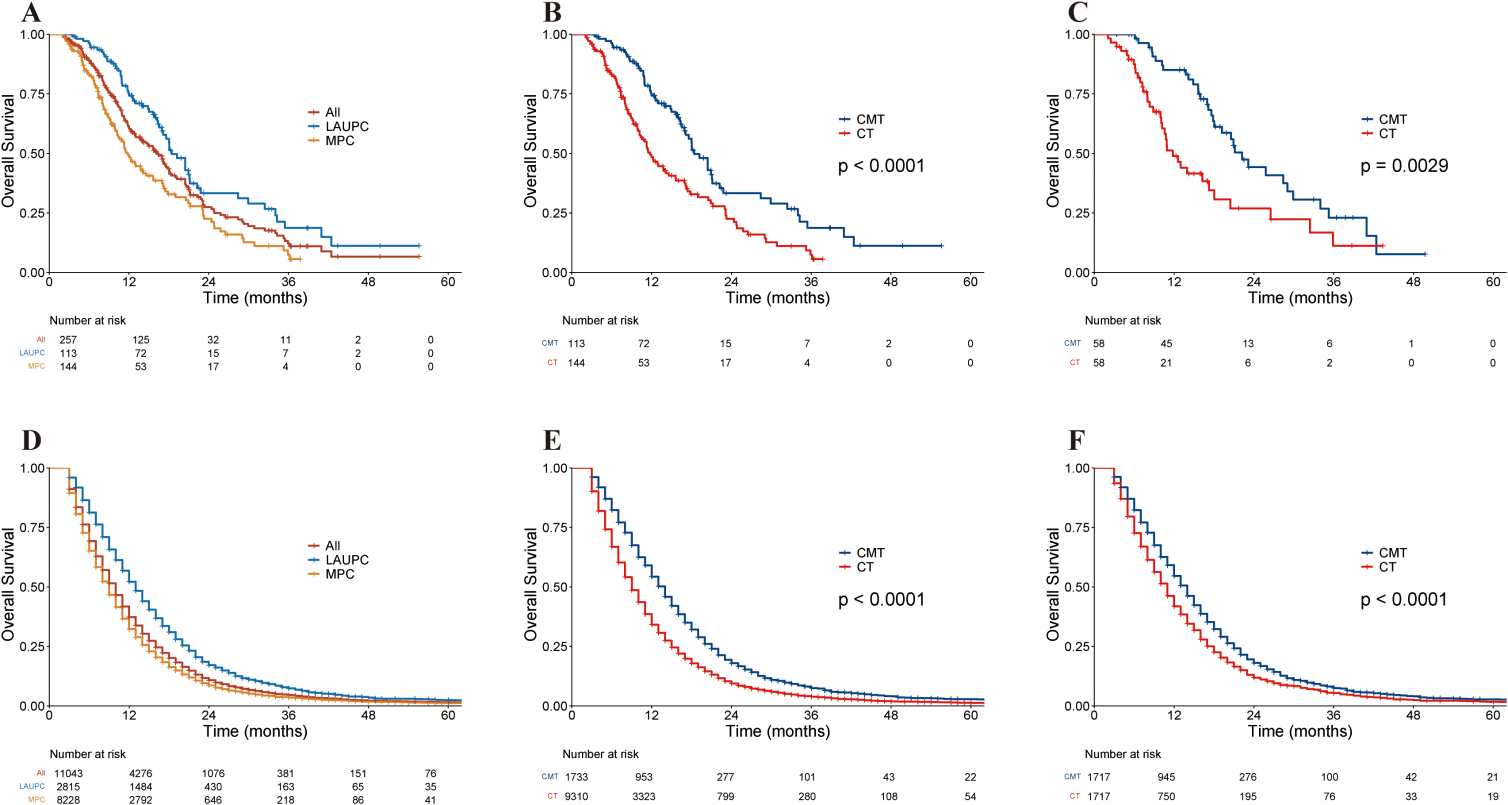
Kaplan–Meier curves of overall survival in the single-institution and the Surveillance, Epidemiology, and End Results (SEER) cohorts. **(A)** Kaplan–Meier curves of overall survival in the single-institution cohort stratified by stage. **(B)** Kaplan–Meier curves of overall survival in the single-institution cohort before PSM, stratified by treatment group. **(C)** Kaplan–Meier curves of overall survival in single-institution cohort after PSM, stratified by treatment group. **(D)** Kaplan–Meier curves of overall survival in the SEER cohort stratified by stage. **(E)** Kaplan–Meier curves of overall survival in the SEER cohort before PSM, stratified by treatment group. **(F)** Kaplan–Meier curves of overall survival in the SEER dataset after PSM, stratified by treatment group. CMT, combined-modality therapy; CT, chemotherapy; LAUPC, locally advanced unresectable pancreatic cancer; MPC, metastatic pancreatic cancer; PSM, propensity-score matching; SEER, Surveillance, Epidemiology, and End Results.

With a median follow-up of 11.8 months (range, 2.1–55.6 months), 158 patients died, 35 patients still alive and 64 were lost to follow-up at the time of analysis. The median OS (mOS) for the entire population was 16.4 months (95% CI, 13.8–18.0 months), with 18.4 months (95% CI, 17.0–22.2 months) for patients with LAUPC and 11.8 months (95% CI, 10.2–15.6 months) for patients with MPC (**Figure 2A**). The corresponding 1- and 2-year OS for the entire population were 60.3% (95% CI, 54.2–67.2%) and 27.5% (95% CI, 21.2–35.6%), respectively.

The median follow-up was 16.0 months (range, 3.4–55.6 months) for the CMT group and 9.3 months (range, 2.1–43.4 months) for the CT group. Before PSM, the mOS was 20.5 months (95% CI, 17.6–25.8 months) in the CMT group and 11.5 months (95% CI, 10.8–13.9 months) in the CT group. The 2-year OS rates were 37.2% (95% CI, 27.2%–51.1%) and 20.4% (95% CI, 13.4%–31.0%) for the CMT and CT groups (p < 0.001) (**Figure 2B**). after PSM. The mOS for the CMT and CT groups was 22.2 months (95% CI, 18.0–34.0 months) and 11.8 months (95% CI, 10.2–20.4 months), respectively, and the 2-year OS was 44.2% (95% CI, 31.2%–62.6%) and 26.8% (95% CI, 15.4%–46.8%) for the CMT and CT groups, respectively (p = 0.003; **Figure 2C**).

#### Prognostic factors

Before PSM, univariable analysis revealed that primary tumor site, T stage, N stage, AJCC stage, presence of liver metastasis, and treatment group were significantly associated with OS. In multivariable analysis, the treatment group was the only significant prognostic factor. After PSM, univariable analysis revealed that primary tumor site, AJCC stage, liver metastasis, and treatment group were significantly associated with OS. In the multivariable analysis, the treatment group was the only significant prognostic factor. Notably, both before PSM (HR: 0.49, 95% CI, 0.30–0.79, p = 0.003) and after PSM (HR: 0.45, 95% CI, 0.27–0.73, p = 0.001), CMT was consistently identified as a significant and independent predictor of OS (**Table 2**).

**Table 2:** Univariable and multivariable Cox regression analyses of prognostic factors for OS in single-institution dataset before and after PSM.

Variables	Before PSM				After PSM			
	HR (univariable)	P	HR (multivariable)	P	HR (univariable)	P	HR (multivariable)	P
Age (years)								
≤55	Reference							
>55	1.21 (0.88-1.67)	0.230			1.17 (0.73-1.90)	0.512		
Sex								
Male	Reference							
Female	1.11 (0.80-1.53)	0.525			0.91 (0.56-1.49)	0.719		
Site								
Head	Reference							
Body/tail	1.49 (1.08-2.05)	0.015	1.23 (0.85-1.77)	0.269	1.69 (1.04-2.77)	0.036	1.64 (0.97-2.76)	0.064
Size (cm)								
≤4	Reference							
>4	1.00 (0.73-1.36)	0.978			1.03 (0.64-1.67)	0.888		
CA19-9 (U/mL)								
≤350	Reference							
>350	1.16 (0.85-1.59)	0.355			1.04 (0.64-1.68)	0.881		
T stage								
T1	Reference							
T2	0.92 (0.46-1.82)	0.804	1.13 (0.55-2.33)	0.731	0.83 (0.18-3.88)	0.817		
T3	0.90 (0.46-1.73)	0.745	0.95 (0.48-1.85)	0.869	1.32 (0.30-5.75)	0.711		
T4	0.49 (0.26-0.93)	0.028	0.78 (0.33-1.80)	0.554	0.67 (0.16-2.80)	0.585		
N stage								
N0	Reference							
N+	1.45 (1.05-2.00)	0.024	1.20 (0.86-1.69)	0.287	0.98 (0.60-1.59)	0.932		
Stage								
III	Reference							
IV	1.90 (1.37-2.62)	p<0.001	0.81 (0.36-1.78)	0.593	1.73 (1.06-2.80)	0.027	1.08 (0.25-4.60)	0.916
Liver metastasis								
No	Reference							
Yes	1.87 (1.36-2.57)	p<0.001	1.46 (0.84-2.54)	0.175	1.76 (1.09-2.86)	0.022	1.52 (0.35-6.64)	0.574
Immunotherapy								
No	Reference							
Yes	0.99 (0.72-1.36)	0.959			0.75 (0.46-1.23)	0.256		
Targeted therapy								
No	Reference							
Yes	0.94 (0.69-1.30)	0.724			0.97 (0.60-1.58)	0.897		
Treatment group								
CT	Reference							
CMT	0.47 (0.34-0.65)	0.001	0.54 (0.38-0.77)	0.001	0.49 (0.30-0.79)	0.003	0.45 (0.27-0.73)	0.001

#### Subgroup analysis

**Figure 3:**
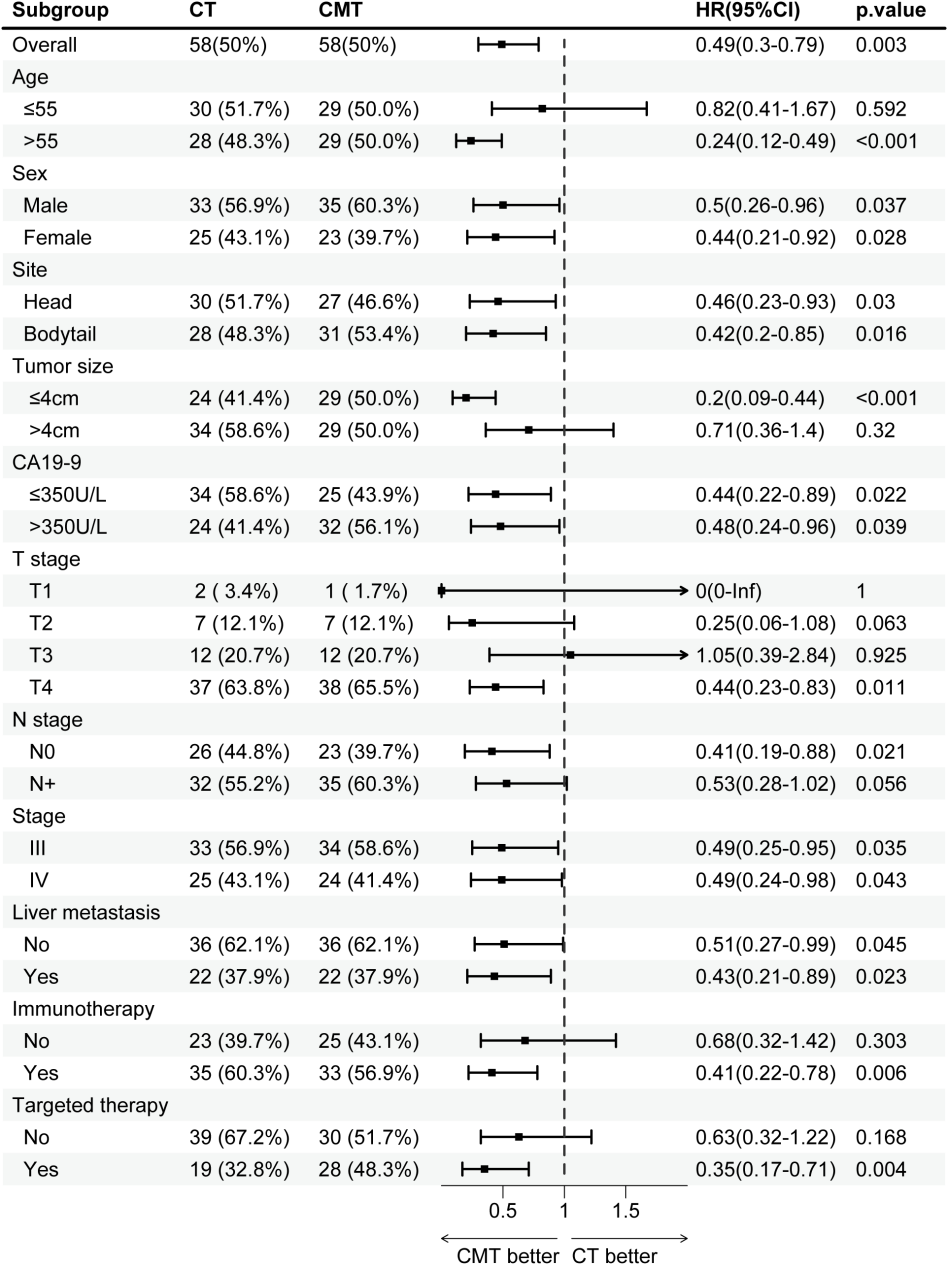
Subgroup analysis of overall survival in the single-institution cohort after PSM, stratified by treatment group. CMT, combined-modality therapy; CT, chemotherapy; PSM, propensity-score matching.

To evaluate the impact of CMT on specific subgroups, subgroup analyses were conducted before and after PSM. Before PSM, CMT benefited patients across majority of subgroups, except for subgroups of T1 (HR, 1.1; 95% CI, 0.31–3.86; p = 0.881) or T3 (HR, 0.82; 95% CI, 0.41–1.63; p = 0.57). The forest plots in **Supplementary Figure S1** visually display the HRs and associated CIs for all subgroups. After PSM, the results illustrated in the forest plots shown in **Figure 3** indicated a significant OS advantage for CMT in most subgroups, except for the following subgroups: age ≤55 years (HR, 0.82; 95% CI, 0.41–1.67; p = 0.592), tumor size >4 cm (HR, 0.71; 95% CI, 0.36–1.40; p = 0.32), T2 (HR, 0.25; 95% CI, 0.06–1.08; p = 0.063), T3 (HR, 1.05; 95% CI, 0.39–2.84; p = 0.925), not receiving immunotherapy (HR, 0.68; 95% CI, 0.32–1.42; p = 0.303), not receiving targeted therapy (HR, 0.63; 95% CI, 0.32–1.22; p = 0.168).

**Figure 4:**
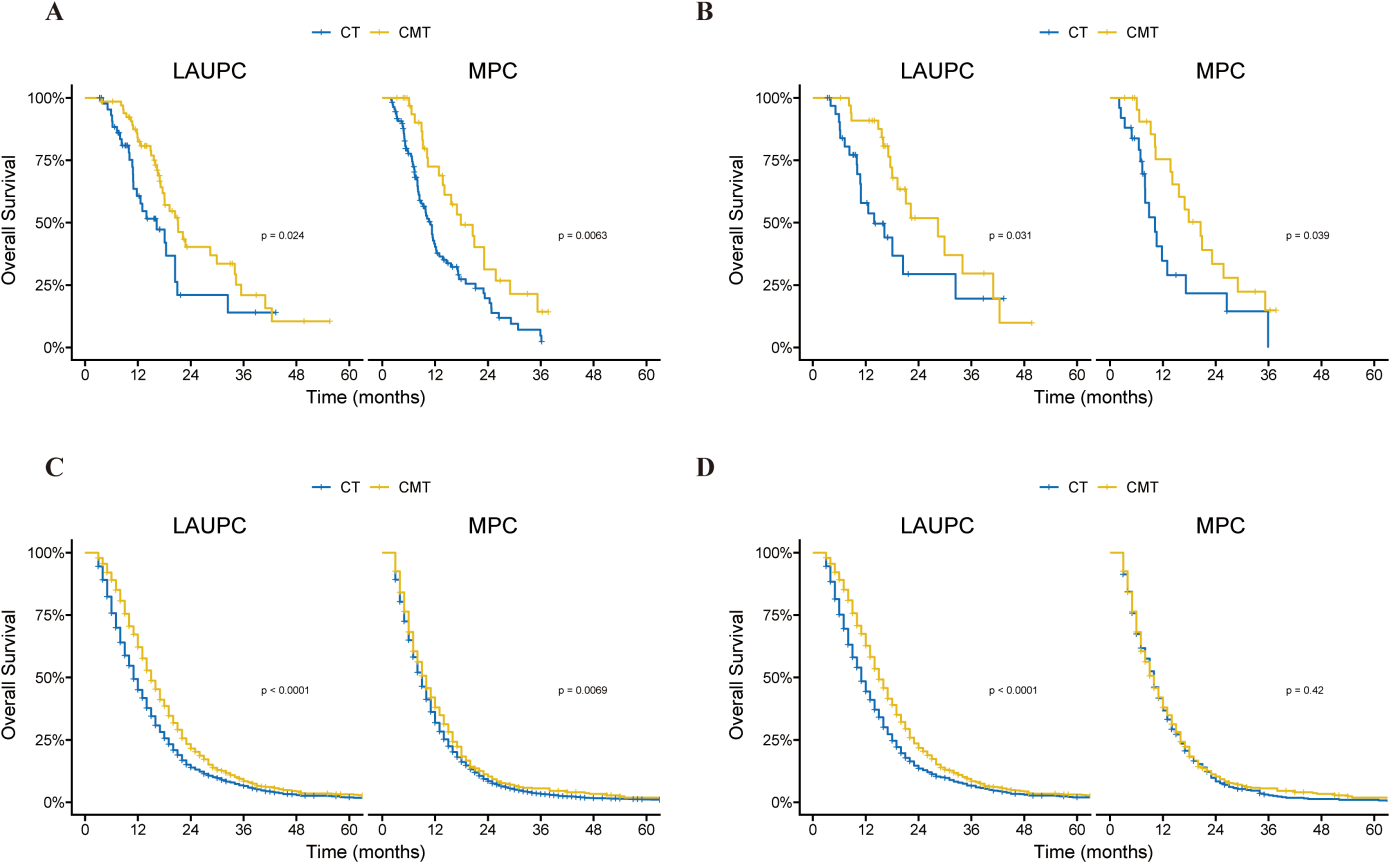
Kaplan–Meier curves of overall survival in the single-institution and SEER cohorts before and after propensity-score matching (PSM), according to treatment group and stage. **(A)** Kaplan–Meier curves of overall survival in the single-institution cohort before PSM, according to treatment group and stage. **(B)** Kaplan–Meier curves of overall survival in the single-institution cohort after PSM, according to treatment group and stage. **(C)** Kaplan–Meier curves of overall survival in the SEER cohort before PSM, according to treatment group and stage. **(D)** Kaplan–Meier curves of overall survival in the SEER cohort after PSM, according to treatment group and stage. CMT, combined-modality therapy; CT, chemotherapy; PSM, propensity-score matching; SEER, Surveillance, Epidemiology, and End Results.

The outcomes of the treatment group were analyzed based on the AJCC stage. Before PSM, in patients with LAUPC, the mOS in the CT group (n=46) and CMT group (n =67) was 16.2 and 21.1 months (p=0.024), respectively (**Figure 4A**). In patients with MPC, the mOS in the CT group (n=109) and CMT group (n=35) were 11.0 months and 17.9 months (p=0.006), respectively (**Figure 4A**). After PSM, the mOS for patients with LAUPC in the CT group (n=33) and CMT group (n=34) was 13.9 and 28.4 months (p=0.031), respectively (**Figure 4B**). The mOS for patients with MPC in the CT group (n=25) and CMT group (n=24) were 10.2 months and 20.5 months (p=0.039), respectively (**Figure 4B**).

### Validation using the SEER cohort

#### Clinicopathologic features

Between January 2010 and December 2020, 11,043 patients with LAUPC and MPC who underwent CMT or CT alone were identified from the SEER database. Before PSM, there were 9,310 patients treated with CT and 1,733 patients treated with CMT. After PSM, there were 1717 patients in each group. The baseline characteristics of the patients before and after PSM are outlined in **Supplementary Table S2**. Before PSM, the CMT group included a higher proportion of patients who were younger, of white race, diagnosed in earlier time periods, had a median income of ≤$55,000, had primary tumor sites in the head, had T4 disease, had stage III disease, and did not have liver metastasis compared to the CT group. All baseline covariates were evenly distributed between the CT and CMT groups after PSM.

#### Oncologic outcomes

The mOS for the entire population was 10.0 months (95% CI, 10.0–10.0 months), with 13.0 months (95% CI, 13.0–14.0 months) for patients with LAUPC and 9.0 months (95% CI, 9.0–9.0 months) for patients with MPC (**Figure 2D**). The corresponding 1-year and 2-year OS rates were 37.3% (95% CI, 36.4–38.3%) and 10.8% (95% CI, 10.2–11.4%), respectively.

Before PSM, the mOS was 14.0 months (95% CI, 13.0–14.0 months) in the CMT group and 9.0 months (95% CI, 9.0–9.0 months) in the CT group before PSM. The 2-year OS rates were 17.9% (95% CI, 16.1–20.0%) and 9.4% (95% CI, 8.8–10.1%) for the CMT and CT groups, respectively (p < 0.001) (**Figure 2E**). After PSM, the mOS for the two groups was 14.0 months (95% CI, 13.0–14.0 months) and 11.0 months (95% CI, 10.0–11.0 months), respectively, and the 2-year OS was 18.1% (95% CI, 16.2–20.1%) and 11.8% (95% CI, 10.3–13.6%) for the CMT and CT groups, respectively (p < 0.001) (**Figure 2F**).

### Prognostic factors

Before PSM, univariable and multivariable analysis revealed that age, race, marital status, year of diagnosis, median income, tumor size, N stage, AJCC stage, liver metastasis, and treatment group were independently significant with OS (**Table 3**). The results of univariable and multivariable analyses after PSM were consistent with those predicted before PSM, except that race was not identified as an independent prognostic factor for OS. CMT was identified as a significantly independent predictor of OS before PSM (HR: 0.82, 95% CI, 0.77–0.87, p < 0.001) and after PSM (HR: 0.80, 95% CI, 0.74–0.86, p < 0.001) (**Table 3**).

**Table 3:** Univariable and multivariable Cox regression analyses of prognostic factors for OS in the SEER dataset before and after PSM.

Variables	Before PSM				After PSM			
	HR (univariable)	P	HR (multivariable)	P	HR (univariable)	P	HR (multivariable)	P
Age (years)								
≤65	Reference							
>65	1.13 (1.09-1.17)	<0.001	1.19 (1.14-1.24)	<0.001	1.10 (1.03-1.18)	0.008	1.17 (1.09-1.26)	<0.001
Sex								
Female	Reference							
Male	1.02 (0.98-1.06)	0.426			0.99 (0.92-1.06)	0.79		
Race								
White	Reference							
Black	1.08 (1.01-1.14)	0.016	1.09 (1.03-1.16)	0.004	0.98 (0.87-1.10)	0.72		
Other	0.91 (0.85-0.98)	0.011	0.99 (0.92-1.06)	0.703	0.89 (0.79-1.00)	0.06		
Marital status								
Married	Reference							
Unmarried	1.11 (1.06-1.16)	<0.001	1.13 (1.08-1.18)	<0.001	1.11 (1.03-1.19)	0.006	1.13 (1.05-1.22)	0.001
Year of diagnosis								
2010-2015	Reference							
2016-2020	0.88 (0.85-0.92)	<0.001	0.87 (0.84-0.91)	<0.001	0.82 (0.76-0.88)	<0.001	0.84 (0.77-0.90)	<0.001
Median income								
≤$55000	Reference							
>$55000	0.87 (0.82-0.91)	<0.001	0.89 (0.85-0.94)	<0.001	0.80 (0.72-0.87)	<0.001	0.83 (0.76-0.91)	<0.001
Site								
Head	Reference							
BodyTail	1.00 (0.96-1.04)	0.908			0.92 (0.85-1.00)	0.042	0.92 (0.85-0.99)	0.035
Other	0.96 (0.91-1.02)	0.177			0.89 (0.81-0.98)	0.017	0.93 (0.84-1.02)	0.128
Tumor size (cm)								
≤4	Reference							
>4	1.08 (1.04-1.13)	<0.001	1.10 (1.05-1.14)	<0.001	1.04 (0.97-1.11)	0.305		
T stage								
T1	Reference							
T2	1.15 (1.02-1.31)	0.022	1.09 (0.97-1.24)	0.157	0.84 (0.57-1.22)	0.357	0.93 (0.63-1.35)	0.69
T3	1.13 (1.00-1.28)	0.048	1.05 (0.92-1.19)	0.457	0.94 (0.65-1.36)	0.735	1.03 (0.71-1.50)	0.883
T4	0.86 (0.76-0.97)	0.014	1.04 (0.91-1.18)	0.596	0.63 (0.44-0.90)	0.011	0.96 (0.66-1.39)	0.825
N stage								
N0	Reference							
N+	1.10 (1.06-1.15)	<0.001	1.09 (1.04-1.13)	<0.001	1.17 (1.09-1.26)	<0.001	1.11 (1.03-1.20)	0.007
Liver metastasis								
No	Reference							
Yes	1.50 (1.44-1.56)	<0.001	1.33 (1.26-1.40)	<0.001	1.59 (1.46-1.74)	<0.001	1.44 (1.27-1.64)	<0.001
Stage								
III	Reference							
IV	1.52 (1.45-1.59)	<0.001	1.13 (1.06-1.22)	0.001	1.44 (1.34-1.56)	<0.001	1.14 (1.00-1.30)	0.046
Treatment group								
CT	Reference							
CMT	0.67 (0.64-0.71)	<0.001	0.82 (0.77-0.87)	<0.001	0.78 (0.73-0.83)	<0.001	0.80 (0.74-0.86)	<0.001

### Subgroup analysis

**Figure 5:**
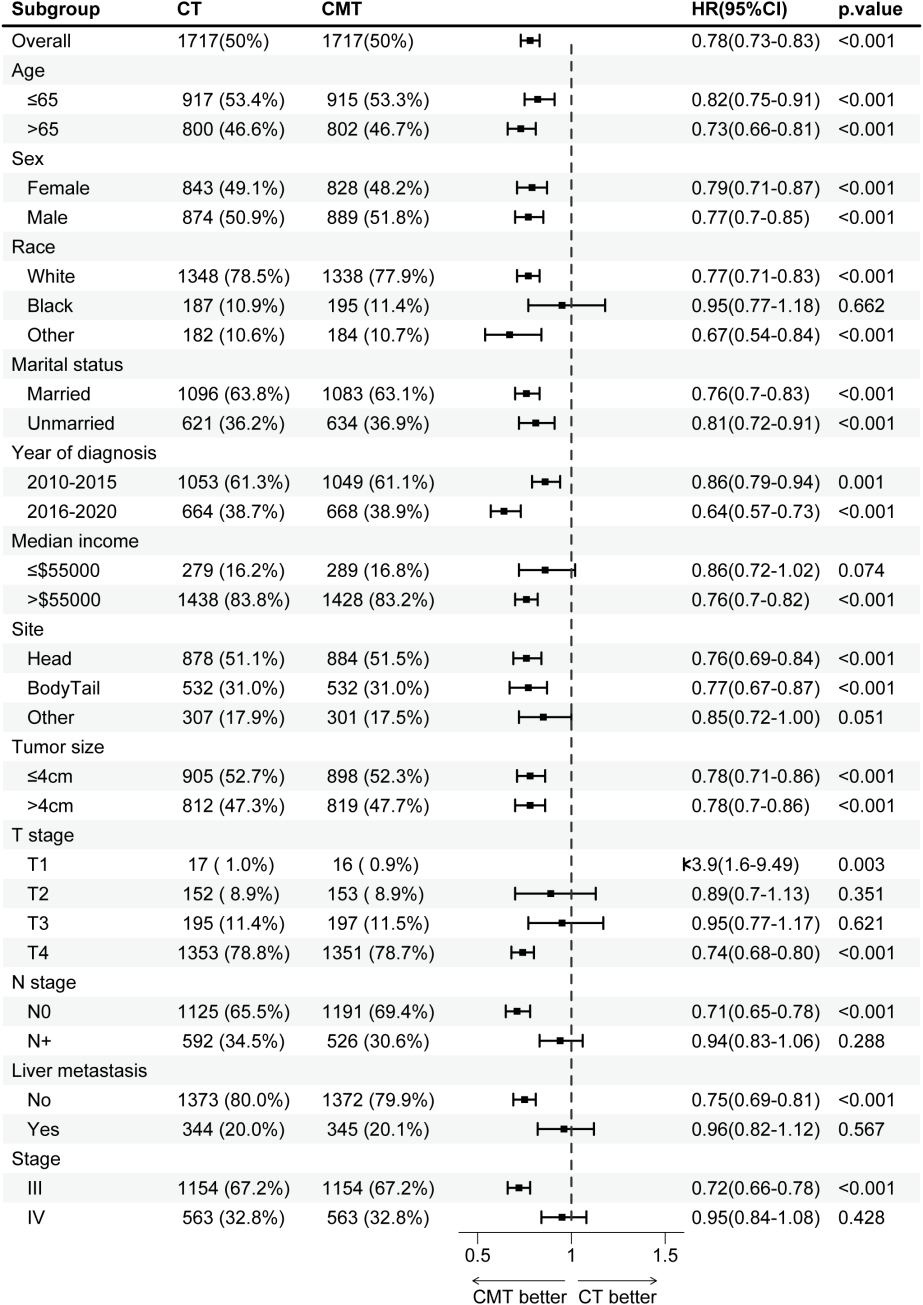
Subgroup analysis of overall survival in the SEER cohort after propensity-score matching (PSM), stratified by treatment group. PSM, propensity-score matching; SEER, Surveillance, Epidemiology, and End Results.

Before PSM, CMT benefited patients in most subgroups, except for subgroups of T3 stage (HR, 0.94; 95% CI, 0.81–1.09; p = 0.421) and liver metastasis (HR, 0.92; 95% CI, 0.82–1.03; p = 0.164). The HRs and associated CIs for all subgroup analyses are graphically summarized in **Supplementary Figure S2**. After PSM, as shown in **Figure 5**, CMT was associated with a significantly increased OS in most subgroups, except for subgroups of black race (HR, 0.95; 95% CI, 0.77–1.18; p = 0.662), median income ≤$55,000 (HR, 0.86; 95% CI, 0.72–1.02; p = 0.074), T2 stage (HR, 0.89; 95% CI, 0.70–1.13; p = 0.351), T3 stage (HR, 0.95; 95% CI, 0.77–1.17; p = 0.621), N+ disease (HR, 0.94; 95% CI, 0.83–1.06; p = 0.288), liver metastasis (HR, 0.96; 95% CI, 0.82–1.12; p = 0.567) and stage IV disease (HR, 0.95; 95% CI, 0.84–1.08; p=0.428).

In patients with LAUPC, the mOS in the CT group (n =1645) and CMT group (n=1170) was 11.0 and 15.0 months (p < 0.001), respectively (**Figure 4C**). In patients with MPC, the mOS in the CT group (n = 7665) and CMT group (n = 563) were 10.0 months and 9.0 months (p = 0.007), respectively (**Figure 4C**). After PSM, the mOS for patients with LAUPC in the CT group (n = 1154) and CMT group (n = 1154) was 11.0 and 15.0 months (p < 0.001), respectively (**Figure 4D**). The mOS for patients with MPC in the CT group (n = 563) and CMT group (n = 563) were 10.0 months and 10.0 months (p = 0.42), respectively (**Figure 4D**).

### Machine learning with XGBoost

**Figure 6:**
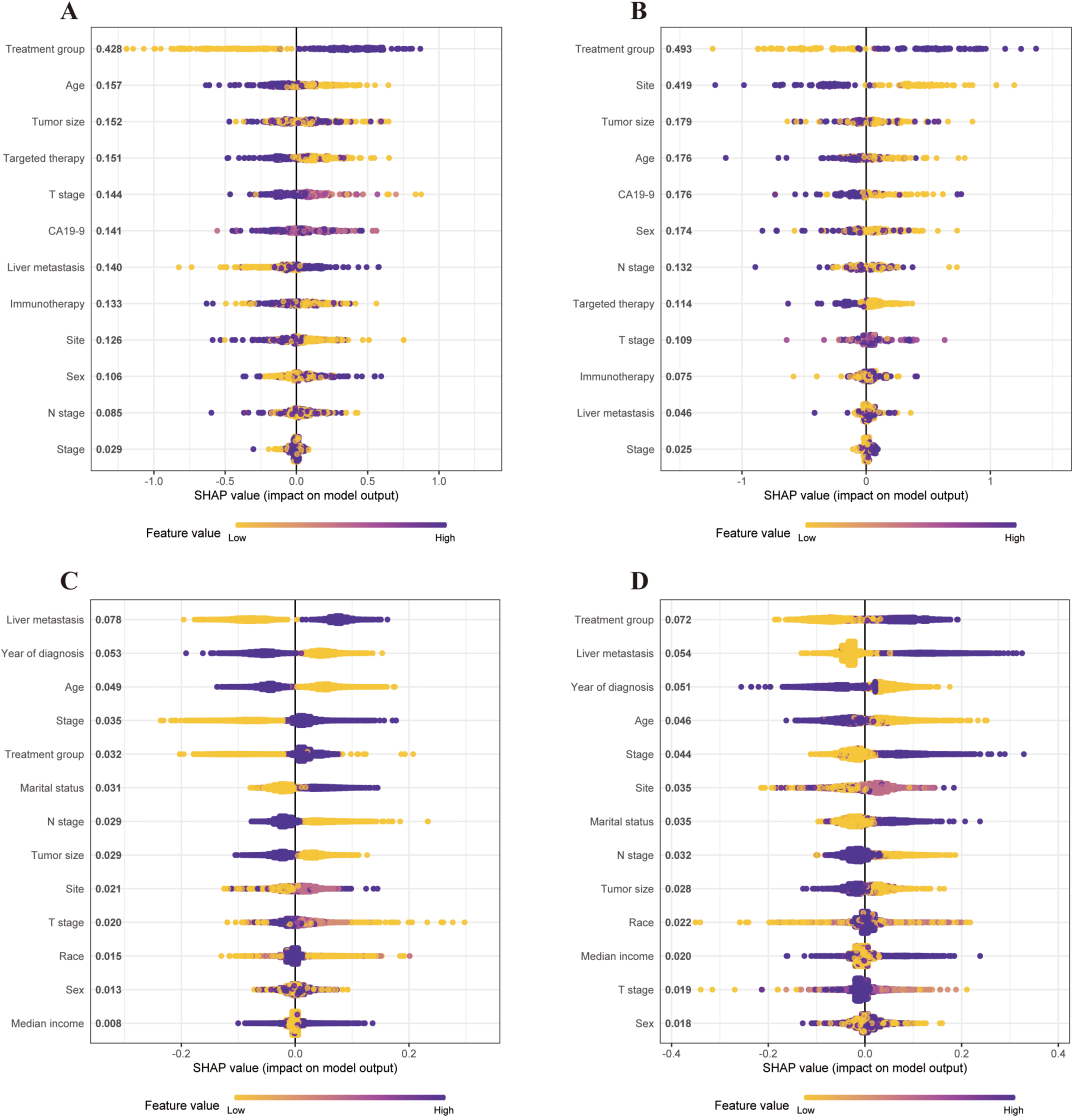
SHAP values of the contribution of each feature to the model output using the single-institution dataset and SEER dataset before and after PSM. Feature importance is represented from top to bottom. **(A)** SHAP values of the contribution of each feature to the model output using the single-institution cohort before PSM. **(B)** SHAP values of the contribution of each feature to the model output using the single-institution cohort after PSM. **(C)** SHAP values of the contribution of each feature to the model output using the SEER cohort before PSM. **(D)** SHAP values of contribution of each feature to the model output using the SEER cohort after PSM. PSM, propensity-score matching; SEER, Statistics, Epidemiology, and End Results; SHAP, Shapley additive explanation.

To elucidate the features of the XGBoost machine learning model, we utilized SHAP graphs to visualize SHAP values for all patients to predict outcomes. In the single-institution cohort, the SHAP algorithm indicated that the treatment group had the greatest impact on predicting outcomes before PSM (**Figure 6A**). This trend persisted after PSM, demonstrating that receiving CMT positively correlated with outcomes and was a protective factor (**Figure 6B**).

In the SEER cohort, liver metastasis had the highest impact on survival predictions before PSM (**Figure 6C**). After PSM, the treatment group emerged as the most significant predictor of survival outcomes in the XGBoost model (**Figure 6D**). Thus, CMT was identified as a key predictor of survival across both datasets.

### Landmark analysis

**Figure 7:**
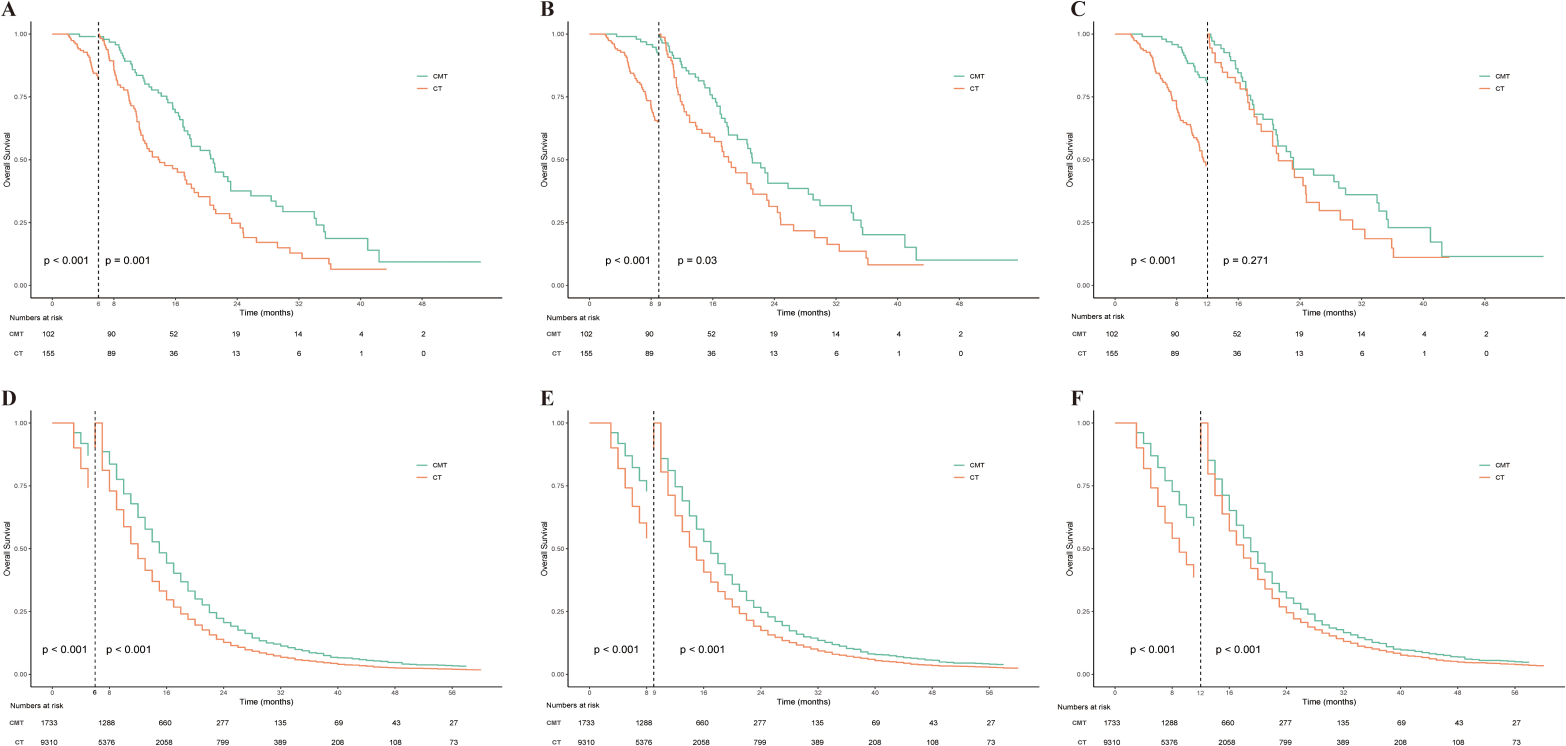
Landmark analysis of OS stratified by treatment group in the single-institution dataset and SEER dataset. **(A)** Landmark analysis of OS stratified by treatment group in the single-institution dataset at 6 months after diagnosis. **(B)** Landmark analysis of OS stratified by treatment group in the single-institution dataset at 9 months after diagnosis. **(C)** Landmark analysis of OS stratified by treatment group in the single-institution dataset at 12 months after diagnosis. **(D)** Landmark analysis of OS stratified by treatment group in the SEER dataset at 6 months after diagnosis. **(E)** Landmark analysis of OS stratified by treatment group in the SEER dataset at 9 months after diagnosis. **(F)** Landmark analysis of OS stratified by treatment group in the SEER dataset at 12 months after diagnosis. OS, overall survival; SEER, Statistics, Epidemiology, and End Results.

We conducted a landmark analysis at 6, 9, and 12 months after diagnosis to reduce immortal time bias. As shown in **Figure 7**, this analysis indicated that CMT could significantly increase overall survival time in patients from both the single-institution and SEER datasets. Notably, in the single-institution data, there was no significant difference in survival between the CMT and CT populations at the 12-month landmark. We speculate this is due to the limited sample size and the small number of subsequent events after the 12-month mark.

## Discussion

In this study, we evaluated the impact of CMT versus CT alone on OS in patients with LAUPC and MPC. Our findings indicate that CMT is associated with improved oncologic outcomes, as demonstrated by longer median OS and lower hazard ratios in both the single-institution cohort and the SEER database validation, both before and after PSM. Multivariate analysis further confirmed the independent association of CMT with enhanced survival outcomes. Several studies have shown the importance of CT in improving survival for LAUPC patients, but there is no consensus on the use of RT. Previous RCTs have yielded inconsistent findings regarding the effects of RT on LAUPC (2–6). Out of the five reviewed RCTs, two found survival benefits of RT for LAUPC (2, 4), while three did not observe any survival benefits (3, 5, 6). The earliest relevant RCT, conducted in 1985, compared 5-fluorouracil (5-FU) alone with 5-FU plus RT, revealing no improvement in overall survival (mOS: 8.2 vs. 8.3 months, p>0.05) (3). In 1988, the Gastrointestinal Tumor Study Group compared streptozotocin, mitomycin C, and 5-FU (SMF) with SMF plus RT, reporting a 1-year OS of 41% and 19% for the CMT and CT groups, respectively (p < 0.02) (2). In 2008, Chaufert et al. (6) investigated 119 patients randomized to either CMT or CT, finding shorter survival in the CMT arm compared to the CT arm (mOS: 8.6 vs. 13 months, p=0.03).Another trial by the Eastern Cooperative Oncology Group randomized 74 patients to receive gemcitabine (GEM) plus RT or GEM alone, revealing significantly prolonged survival in the CMT group (mOS: 11.1 vs. 9.2 months, p =0.017) (4). The LAP07 clinical trial assessed the impact of CMT versus CT on survival in patients following 4 months of gemcitabine treatment with or without erlotinib, finding no significant difference in OS between the two groups (mOS: 15.2 vs. 16.5 months, p = 0.83) (5). Given the variability in outcomes observed in previous RCTs, the application of emerging radiotherapy techniques, such as SBRT, represents a promising enhancement to traditional treatment approaches, potentially offering better local control and survival benefits for LAUPC patients. Parisi et al. (25)presented a promising “COMBO-Therapy” approach combining induction chemotherapy, chemoradiotherapy, and an SBRT boost in patients with LAUPC. Their results demonstrated that adding an SBRT boost, following conventional chemoradiotherapy, significantly improved local control and progression-free survival with a median OS of 21.5 months and a 2-year local control of 72.9%, while maintaining a low toxicity profile. This highlights the potential of dose escalation using SBRT to enhance local tumor control without significant adverse effects. The COMBO-therapy strategy effectively addressed the limitations of traditional RT by delivering higher radiation doses while minimizing toxicity to surrounding organs at risk. In our study, the significant survival benefit observed in patients with LAUPC receiving CMT from our institution is likely attributed to the administration of contemporary chemotherapy protocols and the utilization of more accurate radiotherapy methods in this cohort. Validation results from the SEER dataset further supported these findings. While our findings support the potential benefit of CMT, particularly in improving OS, it is essential to consider advancements in radiotherapy techniques, such as those highlighted by Parisi et al., to optimize therapeutic outcomes further.

As modern precision RT becomes increasingly utilized in the treatment of MPC, the potential to achieve radical tumor control and improve survival outcomes is enhanced (26). RT can synergize with CT to improve disease control and potentially prolong survival, in addition to providing symptomatic relief and improving life quality in patients with MPC (27). Clinical research has demonstrated that combining systemic treatment with local RT targeting all lesions significantly enhances the prognosis of oligometastatic cancer patients (28, 29). In the Stereotactic Ablative Radiotherapy (SABR) for the Comprehensive Treatment of Oligometastases trial, individuals with controlled primary malignancies (such as breast, colorectal, lung, and prostate cancers) and oligometastatic disease who received ablative RT experienced improved progression-free survival (PFS) (mPFS, 12 months vs. 6 months; HR, 0.47; p < 0.01) and OS (mOS, 41 months vs. 28 months; HR, 0.57; p = 0.09) (30). Despite increased toxicity associated with ablative RT, no discernible difference in quality of life was observed. A meta-analysis (31) of 21 trials investigating SABR in patients with oligometastatic cancer, revealed that SABR is generally well-tolerated and provides clinical advantages to this patient population. The rates of acute and late grade 3 to 5 toxic effects were less than 13%, which is considered clinically acceptable. The study found clinically acceptable rates of 1-year local control ranging from 67.2% to 100%, 1-year OS from 65.9% to 100%, and 1-year PFS from 65.9% to 100%. The favorable rates of toxic effects and positive clinical outcomes in terms of local control and survival indicate that SABR is a promising treatment for patients with oligometastatic cancer. A retrospective cohort study on oligometastatic PC highlighted the benefits of SABR for treating all active metastatic sites. The study reported a mOS of 42 months, surpassing that achieved with CT alone (28).

In addition, utilizing local therapy to delay disease progression may provide patients a longer break from conventional CT, reducing cumulative toxicity and improving quality of life. Elamir et al. (28) discovered that definitive RT can postpone the initiation of systemic CT, with 17 out of 20 patients (85%) treated with SABR having a period of 6 months or longer without CT. These findings collectively suggest that CMT is likely to benefit patients with oligometastatic PC. In our study, we observed a favorable OS benefit of CMT for patients with MPC in our institution. However, no survival benefit of CMT for patients with MPC was found in the SEER matched population. This could be due to the more advanced RT techniques used for a subset of MPC patients at our institution, including RT to both metastatic and primary lesions. These findings underscore the potential of CMT as an effective treatment strategy for patients with MPC and warrant further investigation to optimize its utilization in this patient population.

However, while these findings are promising, they must be interpreted with caution due to several critical limitations. Firstly, although the SEER database provided a large sample size, the single-institution cohort was relatively small. This small sample size could introduce bias and affect the robustness of the findings. The potential impact of this limitation on the generalizability of our results is significant, and it suggests that larger, multicenter studies are necessary to confirm these findings and to ensure that they are applicable across different patient populations. Secondly, the SEER database’s lack of detailed information on chemotherapy regimens and radiotherapy (RT) delivery is a notable limitation. These missing details are crucial for a comprehensive comparison of treatment outcomes. Without this information, it is challenging to fully understand how variations in treatment protocols may have influenced the observed survival benefits. This gap in data underscores the need for more granular analyses that include detailed treatment information to draw more definitive conclusions. Additionally, our subgroup analyses revealed significant variability in the effectiveness of CMT across different patient populations. For instance, patients with T3 tumors, younger age, or those not receiving immunotherapy did not show significant benefits from CMT. This suggests that the treatment effect is not uniform and may be influenced by specific patient characteristics. Moreover, some of these subgroup analyses were potentially underpowered due to small sample sizes, which may limit the reliability of these findings. Future studies with larger sample sizes are needed to verify these subgroup differences and to identify which patient populations are most likely to benefit from CMT. Furthermore, the inconsistency observed between the single-institution cohort and the SEER database, particularly regarding the lack of a survival benefit in MPC patients within the SEER database, raises concerns about the universal applicability of CMT. This discrepancy suggests that the effectiveness of CMT may vary depending on patient demographics, clinical characteristics, and treatment protocols. Therefore, a more cautious interpretation of these results is necessary, and further research should focus on understanding the factors contributing to these inconsistencies.

## Conclusion

In conclusion, while our study offers valuable insights into the potential role of CMT in treating LAUPC and MPC, the identified limitations necessitate cautious interpretation. The variability in treatment effects, small single-institution sample size, and lack of detailed treatment data in the SEER database highlight the need for further research. Larger, multicenter studies with comprehensive treatment data are crucial to validate our findings and refine treatment strategies for pancreatic cancer, ultimately improving clinical decision-making.

## Data Availability

The original contributions presented in the study are included in the article/**Supplementary Material**. Further inquiries can be directed to the corresponding authors.
