# Supplementary material for: Survival benefits of radiotherapy in locally advanced unresectable and metastatic pancreatic cancer: a single-institution cohort and SEER database analysis
Source: Front Oncol. 2024 Sep 16;14:1473251. doi: 10.3389/fonc.2024.1473251 (PMC11445777; doi:10.3389/fonc.2024.1473251)

Supplemental Table S1: Detailed information regarding the CT regimens and RT delivery in single-center cohort.

| Chemotherapy^#^ | 257 |
| --- | --- |
| Nab-paclitaxel+S-1 | 90 (35.0%) |
| Nab-paclitaxel+Gemcitabine | 31 (12.1%) |
| FOLFIRINOX | 7 (2.7%) |
| Nab-paclitaxel combination | 8 (3.1%) |
| Gemcitabine combination | 31 (12.1%) |
| Nab-paclitaxel alone | 13 (5.1%) |
| Gemcitabine alone | 19 (7.4%) |
| S-1 alone | 19 (7.4%) |
| Other | 39 (15.2%) |
| Radiotherapy | 102 |
| primary tumor | 76 (74.5%) |
| primary tumor and metastatic lesion | 16 (15.7%) |
| metastatic lesion | 10 (9.8%) |

^# Only regimens in first-line treatment were counted^

Supplemental Table S2: Patient clinicopathologic characteristics by treatment groups in SEER dataset before and after PSM.

| Variables | Before propensity score matching,  n (%) of patients (n = 11043) | | |  | After propensity score matching,  n (%) of patients (n = 3434) | | |
| --- | --- | --- | --- | --- | --- | --- | --- |
|  | CT | CMT | p |  | CT | CMT | p |
| n | 9310 (84.3) | 1733 (15.7) |  |  | 1717 (50) | 1717 (50) |  |
| Age (years) |  |  |  |  |  |  |  |
| ≤65 | 4704 (50.5) | 930 (53.7) | 0.018 |  | 917 (53.4) | 915 (53.3) | 0.973 |
| >65 | 4606 (49.5) | 803 (46.3) |  |  | 800 (46.6) | 802 (46.7) |  |
| Sex |  |  |  |  |  |  |  |
| Female | 4400 (47.3) | 837 (48.3) | 0.443 |  | 843 (49.1) | 828 (48.2) | 0.633 |
| Male | 4910 (52.7) | 896 (51.7) |  |  | 874 (50.9) | 889 (51.8) |  |
| Race |  |  |  |  |  |  |  |
| White | 7388 (79.4) | 1342 (77.4) | 0.033 |  | 1348 (78.5) | 1338 (77.9) | 0.898 |
| Black | 1114 (12.0) | 207 (11.9) |  |  | 187 (10.9) | 195 (11.4) |  |
| Other | 808 (8.7) | 184 (10.6) |  |  | 182 (10.6) | 184 (10.7) |  |
| Marital status |  |  |  |  |  |  |  |
| Married | 5979 (64.2) | 1089 (62.8) | 0.283 |  | 1096 (63.8) | 1083 (63.1) | 0.671 |
| Unmarried | 3331 (35.8) | 644 (37.2) |  |  | 621 (36.2) | 634 (36.9) |  |
| Year of diagnosis |  |  |  |  |  |  |  |
| 2010-2015 | 4886 (52.5) | 1064 (61.4) | <0.001 |  | 1053 (61.3) | 1049 (61.1) | 0.916 |
| 2016-2020 | 4424 (47.5) | 669 (38.6) |  |  | 664 (38.7) | 668 (38.9) |  |
| Median income |  |  |  |  |  |  |  |
| ≤$55000 | 1430 (15.4) | 303 (17.5) | 0.028 |  | 279 (16.2) | 289 (16.8) | 0.679 |
| >$55000 | 7880 (84.6) | 1430 (82.5) |  |  | 1438 (83.8) | 1428 (83.2) |  |
| Primary site |  |  |  |  |  |  |  |
| Head | 3935 (42.3) | 891 (51.4) | <0.001 |  | 878 (51.1) | 884 (51.5) | 0.961 |
| Body & Tail | 3698 (39.7) | 536 (30.9) |  |  | 532 (31.0) | 532 (31.0) |  |
| Other | 1677 (18.0) | 306 (17.7) |  |  | 307 (17.9) | 301 (17.5) |  |
| Tumor size (cm) |  |  |  |  |  |  |  |
| ≤4 | 4892 (52.5) | 904 (52.2) | 0.79 |  | 905 (52.7) | 898 (52.3) | 0.838 |
| >4 | 4418 (47.5) | 829 (47.8) |  |  | 812 (47.3) | 819 (47.7) |  |
| T stage |  |  |  |  |  |  |  |
| T1 | 302 (3.2) | 16 (0.9) | <0.001 |  | 17 (1.0) | 16 (0.9) | 0.997 |
| T2 | 2872 (30.8) | 153 (8.8) |  |  | 152 (8.9) | 153 (8.9) |  |
| T3 | 2624 (28.2) | 198 (11.4) |  |  | 195 (11.4) | 197 (11.5) |  |
| T4 | 3512 (37.7) | 1366 (78.8) |  |  | 1353 (78.8) | 1351 (78.7) |  |
| N stage |  |  |  |  |  |  |  |
| N0 | 6286 (67.5) | 1201 (69.3) | 0.153 |  | 1125 (65.5) | 1191 (69.4) | 0.018 |
| N+ | 3024 (32.5) | 532 (30.7) |  |  | 592 (34.5) | 526 (30.6) |  |
| Liver metastasis |  |  |  |  |  |  |  |
| No | 3441 (37.0) | 1388 (80.1) | <0.001 |  | 1373 (80.0) | 1372 (79.9) | 1 |
| Yes | 5869 (63.0) | 345 (19.9) |  |  | 344 (20.0) | 345 (20.1) |  |
| Stage |  |  |  |  |  |  |  |
| III | 1645 (17.7) | 1170 (67.5) | <0.001 |  | 1154 (67.2) | 1154 (67.2) | 1 |
| IV | 7665 (82.3) | 563 (32.5) |  |  | 563 (32.8) | 563 (32.8) |  |

Supplemental fig. S1: Subgroup analysis of overall survival in the single-institution cohort before PSM stratified by treatment group.


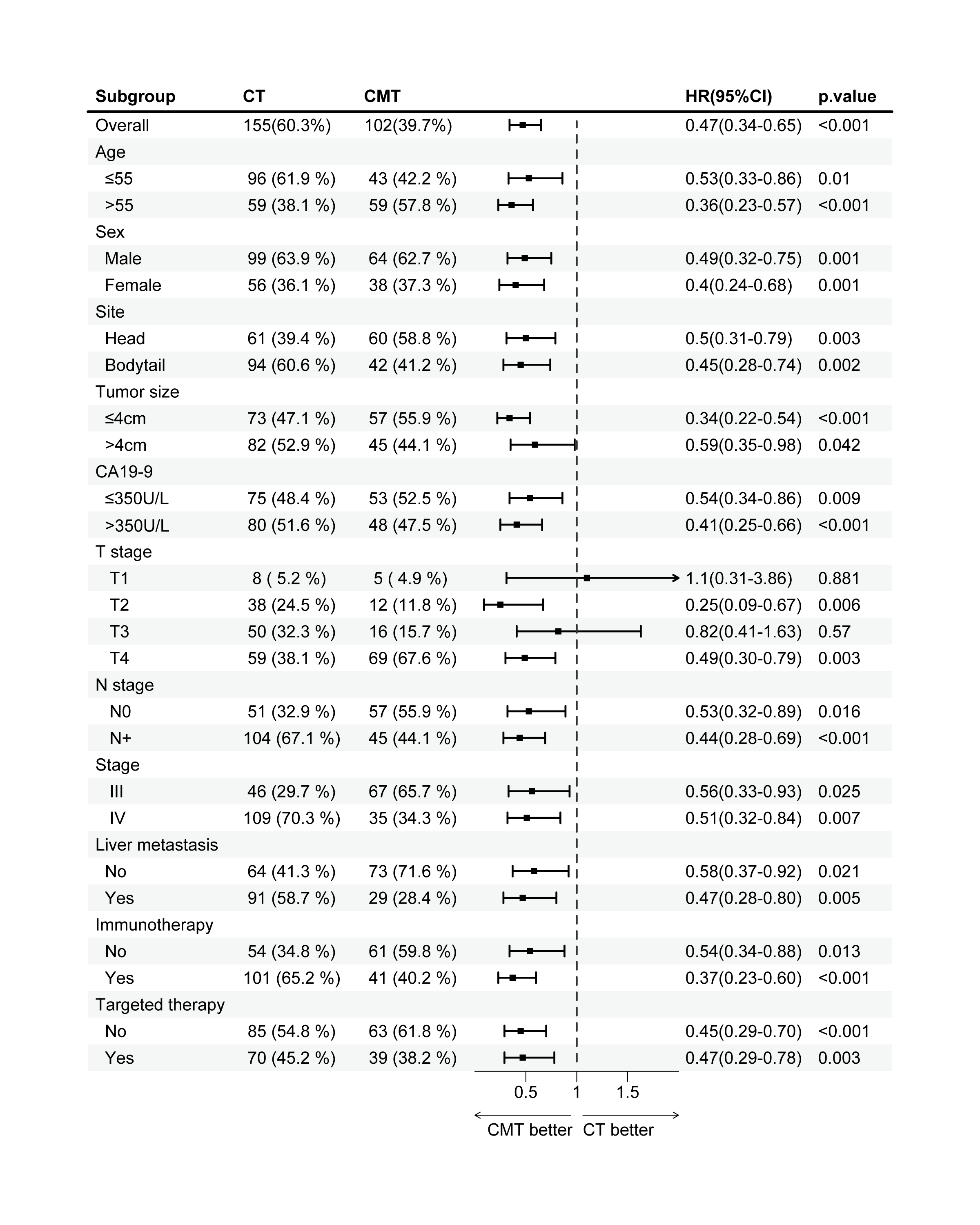


Supplemental fig. S2: Subgroup analysis of overall survival in the SEER cohort before PSM, stratified by treatment group.


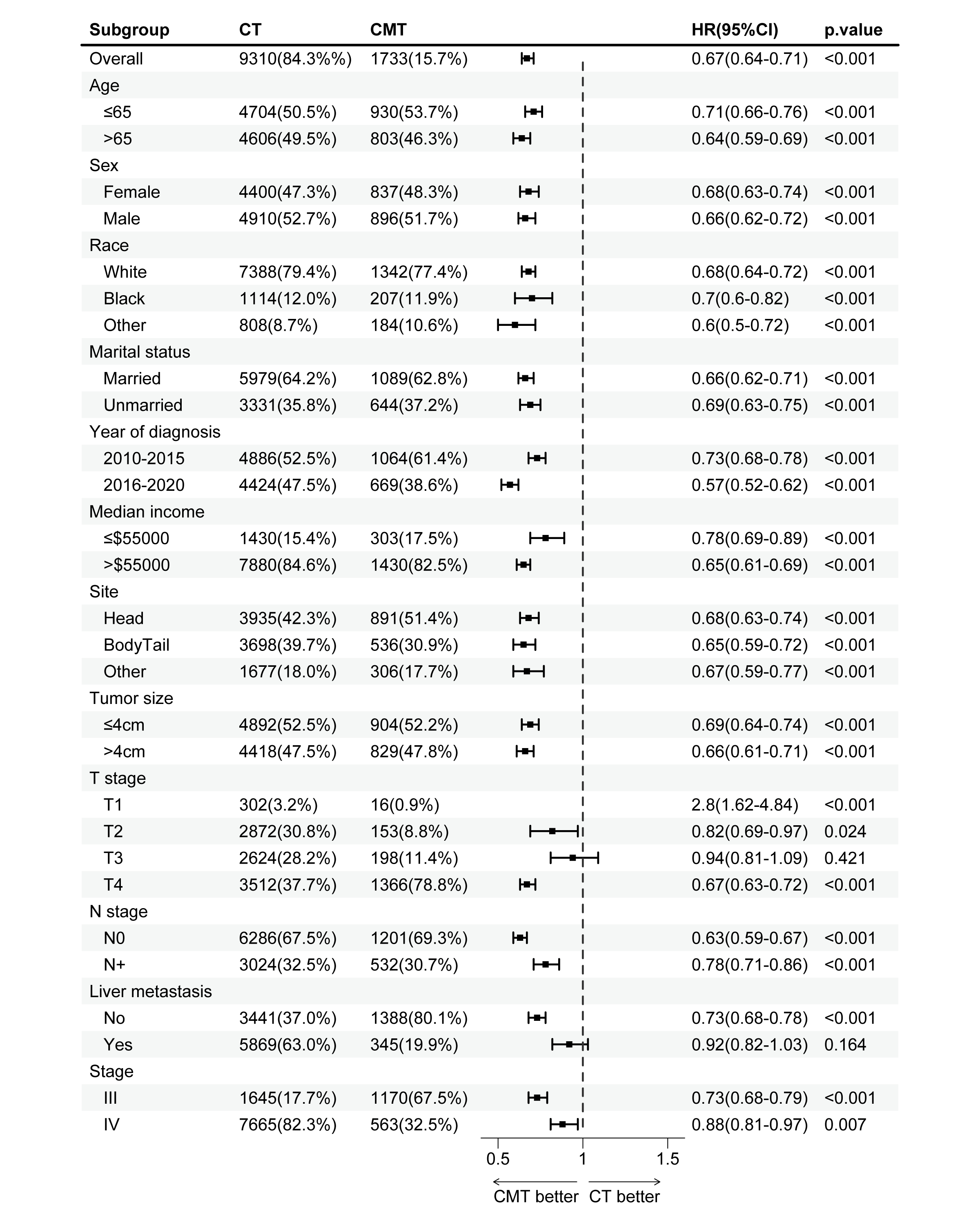

Supplement: Supplementary file 1 [file DataSheet1.docx]
